# A novel CT scoring method predicts the prognosis of interstitial lung disease associated with anti-MDA5 positive dermatomyositis

**DOI:** 10.1038/s41598-021-96292-w

**Published:** 2021-08-23

**Authors:** Wenwen Xu, Wanlong Wu, Danting Zhang, Zhiwei Chen, Xinwei Tao, Jiangfeng Zhao, Kaiwen Wang, Xiaodong Wang, Yu Zheng, Shuang Ye

**Affiliations:** 1 Department of Rheumatology, Shanghai Jiao Tong University School of Medicine Affiliated Renji Hospital, No. 2000 Jiangyue Road, Shanghai, 201112 China; 2 CT Scientific Collaboration, Siemens Healthineers, Shanghai, China; 3 Department of Pulmonology, Shanghai Jiao Tong University School of Medicine Affiliated Renji Hospital, No. 2000 Jiangyue Road, Shanghai, 201112 China

**Keywords:** Idiopathic inflammatory myopathies, Risk factors

## Abstract

Anti-melanoma differentiation-associated gene 5-positive dermatomyositis-associated interstitial lung disease (MDA5^+^ DM-ILD) is a life-threatening disease. This study aimed to develop a novel pulmonary CT visual scoring method for assessing the prognosis of the disease, and an artificial intelligence (AI) algorithm-based analysis and an idiopathic pulmonary fibrosis (IPF)-based scoring were conducted as comparators. A retrospective cohort of hospitalized patients with MDA5^+^ DM-ILD was analyzed. Since most fatalities occur within the first half year of the disease course, the primary outcome was the six-month all-cause mortality since the time of admission. A ground glass opacity (GGO) and consolidation-weighted CT visual scoring model for MDA5^+^ DM-ILD, namely ‘MDA5 score’, was then developed with C-index values of 0.80 (95%CI 0.75–0.86) in the derivation dataset (n = 116) and 0.84 (95%CI 0.71–0.97) in the validation dataset (n = 57), respectively. While, the AI algorithm-based analysis, namely ‘AI score’, yielded C-index 0.78 (95%CI 0.72–0.84) for the derivation dataset and 0.77 (95%CI 0.64–0.90) for the validation dataset. These findings suggest that the newly derived ‘MDA5 score’ may serve as an applicable prognostic predictor for MDA5^+^ DM-ILD and facilitate further clinical trial design. The AI based CT quantitative analysis provided a promising solution for ILD evaluation.

## Introduction

Anti-melanoma differentiation-associated gene 5-positive dermatomyositis (MDA5^+^ DM) is a special subtype of DM, associated with rapidly progressive interstitial lung disease (ILD). The overall prognosis is grave with 33–67% six-month mortality despite of aggressive immunosuppressive therapy^1–4^.

Pulmonary high-resolution computed tomography (HRCT) is a main-stream imaging tool for identifying ILD and measuring its severity. A semi-quantitative HRCT scoring system has been applied as a prognostic prediction measurement in MDA5^+^ DM-ILD^5, 6^. However, the applied scoring system was initially designed for evaluation of idiopathic pulmonary fibrosis (IPF)^7, 8^. Therefore, when referring to a more rapid progressive disease, such as MDA5^+^ DM-ILD, the applicability has not been extensively validated. As examples, fibrosis components such as traction bronchiectasis (TBE) and honeycombing changes were higher weighted in this ‘IPF score’; whereas inflammation components, i.e., ground-glass opacity (GGO) and consolidation were less weighted. Only until recently, another simplified scoring method for MDA5^+^ DM-ILD was proposed with equally weighted two components of GGO and fibrosis^9, 10^. Unfortunately, the sample size was small with characteristic consolidation feature being overlooked; further independent evaluation in a data-driven approach is warranted. It is noteworthy that the time-consuming observer-dependent manner of these visual scorings is always an issue.

Under the pressure of the coronavirus disease 2019 (COVID-19) pandemic, advanced machine learning-based technologies on pulmonary CT quantitative analysis have rapidly emerged, providing a promising solution for diffuse lung disease HRCT evaluation in a more comprehensive and objective perspective^11–13^.

Thus, the aims of the current study were to establish a novel pulmonary HRCT visual scoring method for predicting the six-month mortality in a large single-centered cohort of patients with MDA5^+^ DM; and in parallel, to explore quantitative imaging assessment of this disease by applying artificial intelligence (AI) algorithm.

## Results

Comparable baseline clinical features, treatment and outcomes of the derivation dataset and validation dataset were listed in Supplementary table S1. Of which, 47 (40.5%) and 21 (36.8%) patients died within six-month follow up since the time of admission, respectively (p = 0.764).

### ‘MDA5 score’: a novel CT visual semi-quantitative analysis

The pulmonary HRCT findings from visual semi-quantitative analysis of patients between survivors and non-survivors in both datasets were presented in Table 1. As expected, the ILD pattern distributed bilaterally. It was noteworthy that only GGO and consolidation patterns were significantly associated with outcome according to univariable analysis; as opposed to neither fibrosis nor the presence of pneumomediastinum or pneumothorax (PNM) at baseline. Then, the GGO and consolidation score were included in further multivariable COX regression analysis. Both total GGO score (β coefficient = 0.13, *p* < 0.001) and total consolidation score (β coefficient = 0.22, *p* < 0.001) were determined to be significantly associated with all-cause mortality (Table 2). To simplify, a linear equation, namely ‘MDA5 score’, by combining defined prognostic factors weighted by their β coefficients was finally generated: total GGO score + 2*total consolidation score.

**Table 1:** Comparison of visual CT features between two datasets with different outcome.

	Derivation dataset			p-value*	Validation dataset			p-value*	p-value^**†**^
	All (n = 116)	Survivors (n = 69)	Non-survivors (n = 47)		All (n = 57)	Survivors (n = 36)	Non-survivors (n = 21)		
**Distribution pattern**									
Bilateral lung involved	113 (97.4)	66 (95.7)	47 (100.0)	0.39	54 (94.7)	33 (91.7)	21 (100.0)	0.46	0.64
RU lobe involved	95 (81.9)	50 (72.5)	45 (95.7)	**0.003**	48 (84.2)	27 (75.0)	21 (100.0)	**0.03**	0.87
RM lobe involved	76 (65.5)	36 (52.2)	40 (85.1)	**0.001**	42 (73.7)	22 (61.1)	20 (95.2)	**0.01**	0.36
RL lobe involved	113 (97.4)	66 (95.7)	47 (100.0)	0.39	57 (100.0)	36 (100.0)	21 (100.0)	1	0.55
LU lobe involved	99 (85.3)	54 (78.3)	45 (95.7)	**0.02**	51 (89.5)	30 (83.3)	21 (100.0)	0.13	0.61
LL lobe involved	113 (97.4)	67 (97.1)	46 (97.9)	1	54 (94.7)	33 (91.7)	21 (100.0)	0.46	0.64
No. involved lobe				**0.001**				**0.02**	0.17
1	2 (1.7)	2 (2.9)	0 (0.0)		3 (5.3)	3 (8.3)	0 (0.0)		
2	12 (10.3)	11 (15.9)	1 (2.1)		1 (1.8)	1 (2.8)	0 (0.0)		
3	7 (6.0)	5 (7.2)	2 (4.3)		4 (7.0)	4 (11.1)	0 (0.0)		
4	26 (22.4)	21 (30.4)	5 (10.6)		10 (17.5)	9 (25.0)	1 (4.8)		
5	69 (59.5)	30 (43.5)	39 (83.0)		39 (68.4)	19 (52.8)	20 (95.2)		
No. involved lobes > 3	95 (81.9)	51 (73.9)	44 (93.6)	**0.01**	49 (86.0)	28 (77.8)	21 (100.0)	0.05	0.65
No. involved lobes > 4	69 (59.5)	30 (43.5)	39 (83.0)	** < 0.001**	39 (68.4)	19 (52.8)	20 (95.2)	**0.002**	0.33
PNM	5 (4.3)	2 (2.9)	3 (6.4)	0.39	3	1 (2.8)	2 (9.5)	0.55	0.72
**Scoring of three components**									
Total GGO score	4 [2–8]	3 2–6]	7 [3–10]	** < 0.001**	6 [2–11]	5 [1–8]	10 [7–12]	**0.002**	0.13
Total CON score	4 [2–8]	3 [15]	8 [5–11]	** < 0.001**	6 4–9]	5 [2–7]	9 [7–13]	** < 0.001**	0.06
Total fibrosis score	3 [2–5]	2 [2–4]	3 [2–5]	0.51	4 [2–5]	4 [2–5]	5 [2–5]	0.51	0.01
**IPF score**	126 [116–151]	118 [112–127]	147 [125–171]	** < 0.001**	135 [121–155]	127 [112–141]	153 [134–188]	** < 0.001**	0.12

Data are presented as median [IQR] for continuous variables and number (frequency) (%) for categorical variables.

* A comparison between the survivors and non-survivors groups.

^**†**^ A comparison between the derivation and validation datasets.

CT, computed tomography; RU, right upper; RM, right middle; RL, right lower; LU, left upper; LL, left lower; PNM, pneumomediastinum or pneumothorax; GGO, ground-glass opacity; CON, consolidation; IPF score, idiopathic pulmonary fibrosis based visual scoring method.

**Table 2:** Multivariable COX regression analysis for ‘MDA5 score’ and ‘AI score’ models.

	HR [95%CI]	P-value	β coefficient
**‘MDA5 score’ model**			
Total GGO score	1.14 [1.06–1.22]	< 0.001	0.13
Total consolidation score	1.25 [1.16–1.34]	< 0.001	0.22
**‘AI score’ model**			
Percentage of GGO	1.02 [1.00, 1.04]	0.07	0.02
Percentage of consolidation	1.15 [1.08, 1.22]	< 0.001	0.14

HR, hazard ratio; 95%CI, 95% confidence interval; MDA5, anti-melanoma differentiation-associated gene 5; AI, artificial intelligence; GGO, ground-glass opacity.

**Figure 1:**
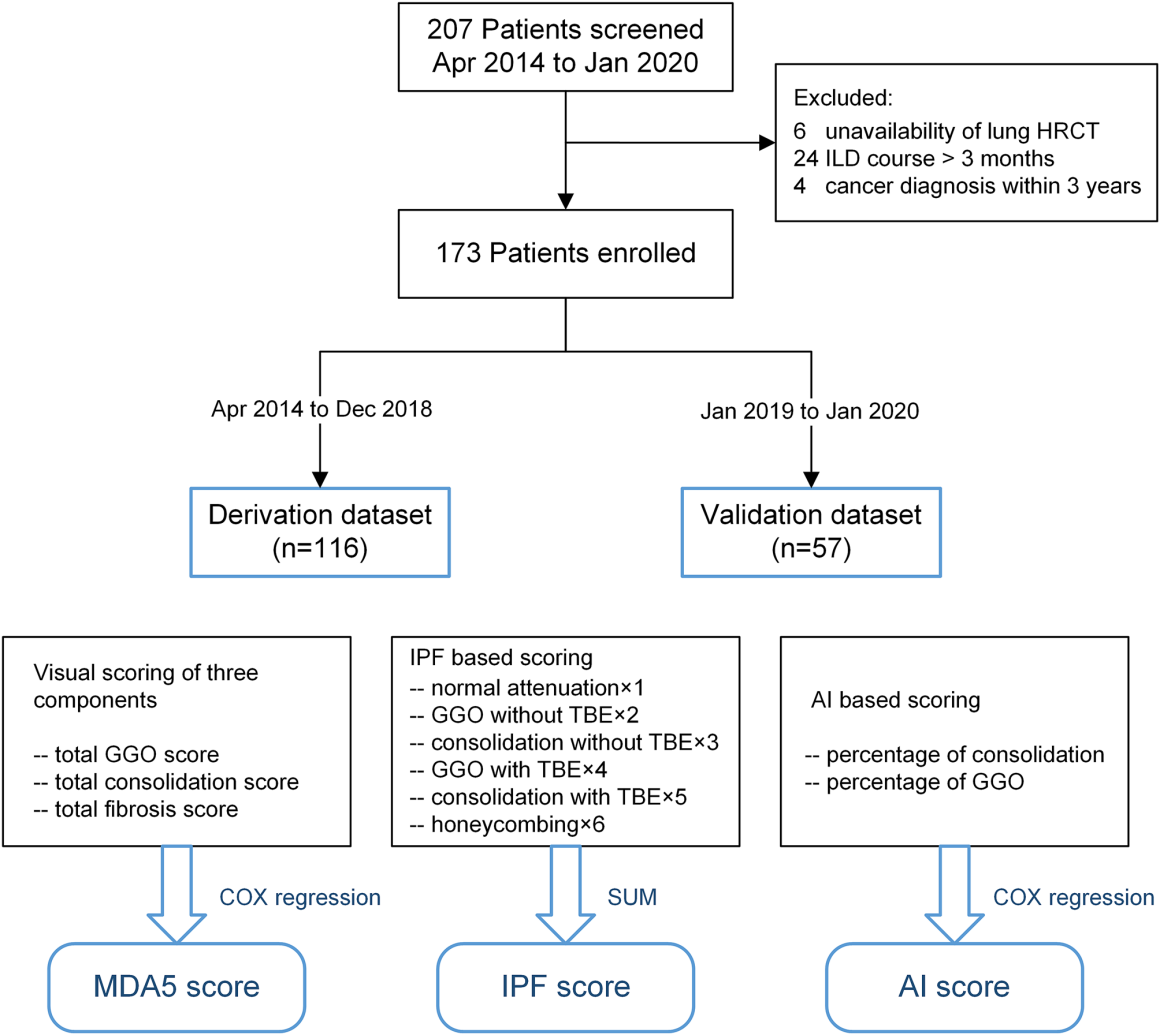
Flow chart of patients and three CT scoring models. MDA5, melanoma differentiation-associated gene 5; HRCT, high-resolution computed tomography; ILD, interstitial lung disease; GGO, ground-glass opacity; TBE, traction bronchiectasis; IPF, idiopathic pulmonary fibrosis; AI, artificial intelligence; DM, dermatomyositis.

**Figure 2:**
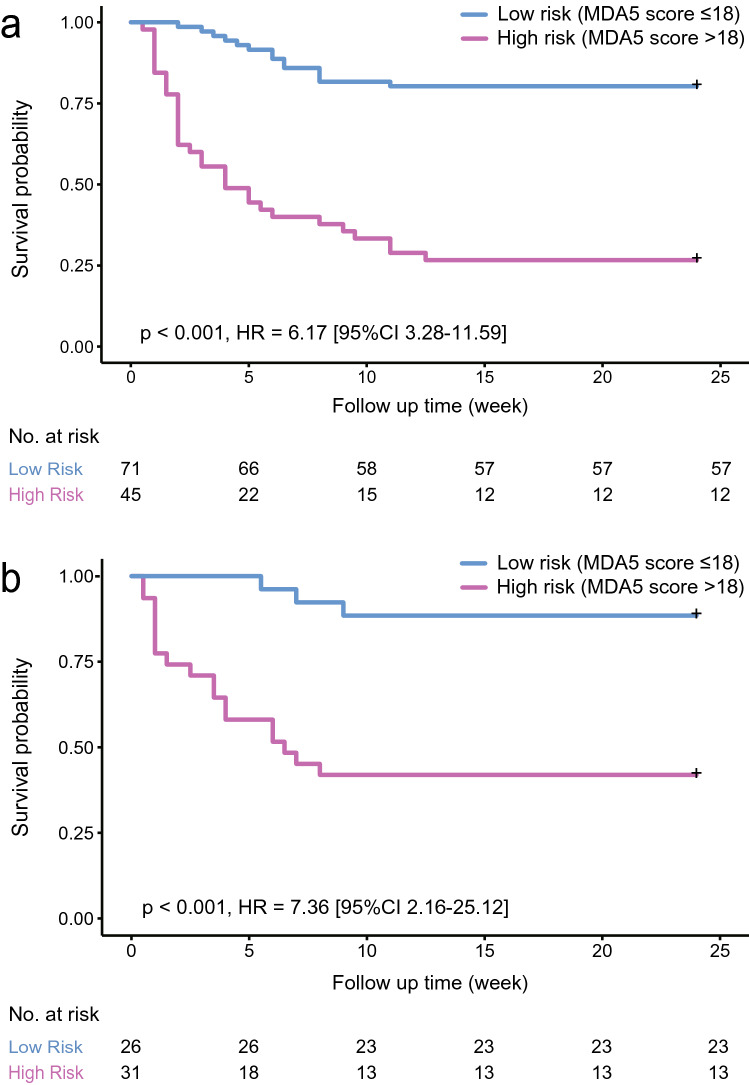
Survival curves of ‘MDA5 score’ in the derivation (**a**) and validation (**b**) datasets. MDA5, melanoma differentiation-associated gene 5; HR, hazard ratio; 95%CI, 95% confidence interval.

ROC curve analysis indicated that the optimal cutoff value for ‘MDA5 score’ was 18, which could efficiently predict the six-month all-cause mortality in the derivation dataset (sensitivity 70.2%; specificity 82.6%) and the validation dataset (sensitivity 85.7%; specificity 63.9%). The prediction accuracy of ‘MDA5 score’ calculated by AUC was 0.85 (95%CI 0.78–0.91) for the derivation dataset and 0.87 (95%CI 0.78–0.96) for the validation dataset, far ahead of the ‘IPF score’, which was 0.81 (95%CI 0.73–0.89) for the derivation dataset and 0.79 (95%CI 0.68–0.91) for the validation dataset. Additionally, The Kaplan–Meier survival plots of patients in both datasets presented significant difference between the high-risk (‘MDA5 score’ > 18) and low-risk (‘MDA5 score’ ≤ 18) groups (Figs. 1, 2). The mortality of high-risk patients was 73.3% in the derivation dataset and 58.1% in the validation dataset; while the mortality of low-risk patients was 19.7% in the derivation dataset and 11.5% in the validation dataset.

### ‘AI score’: an AI algorithm-based quantitative analysis

**Figure 3:**
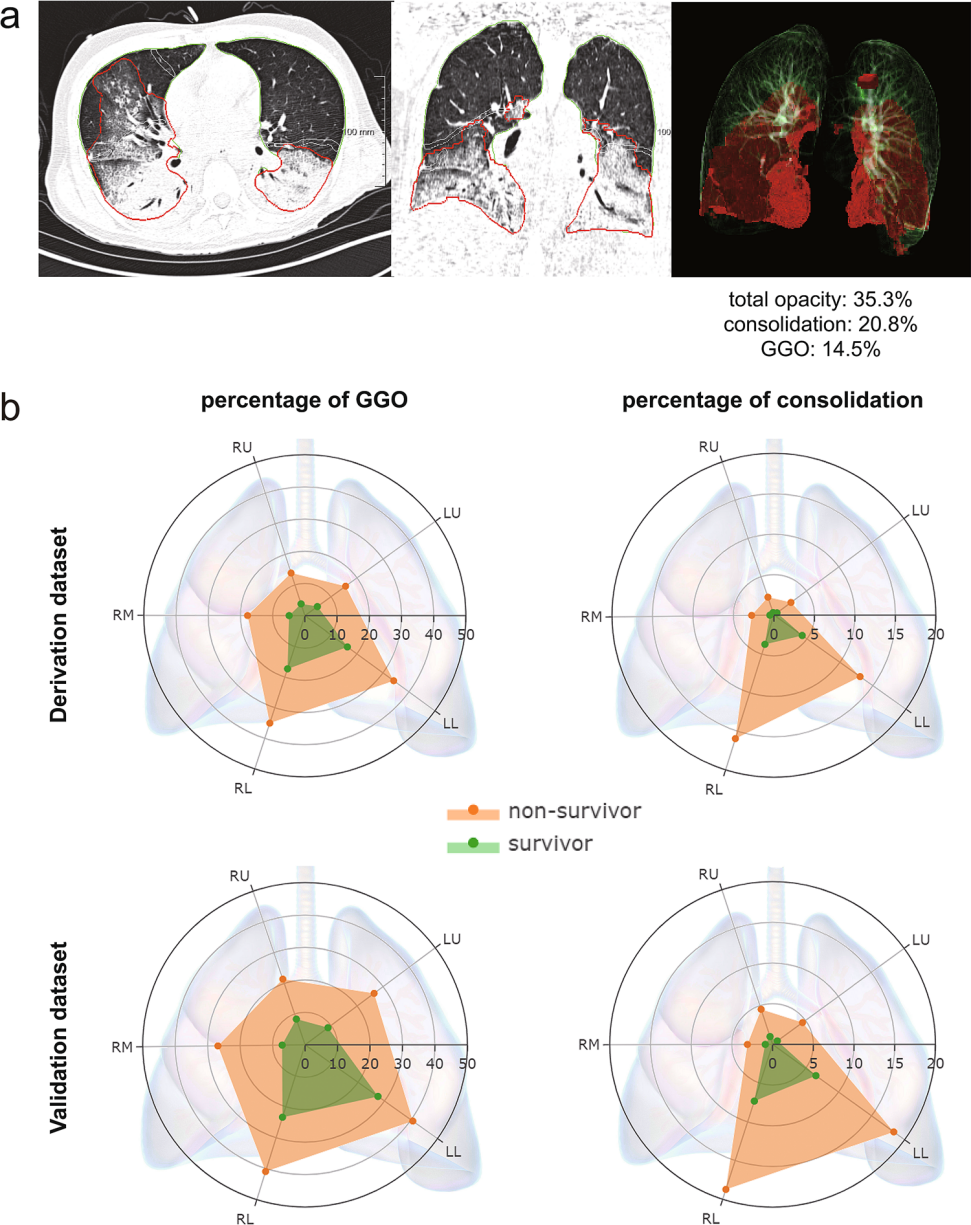
Artificial intelligence algorithm-based CT quantitative analysis. (**a**) The segmentation results of the lung and its total opacity in representative CT images were shown in green and red borders respectively. The percentage of total opacity, consolidation, and ground-glass opacity (GGO) of the whole lung were automatically calculated to be 35.3%, 20.8%, and 14.5% respectively. (**b**) The average distributions of GGO and consolidation in each lobe of the lung were displayed by the radar charts in both datasets. The axial line of the radar chart referred to the mean percentage (%) of either GGO or consolidation of each lobe. RU, right upper lobe; RM, right middle lobe; RL, right lower lobe; LU, left upper lobe; LL, left lower lobe.

The redundancy of the baseline fibrosis component in terms of outcome prediction made pneumonia-trained AI algorithm plausible for our MDA5^+^ DM-ILD patients’ CT quantitative analysis (Fig. 3A). Percentage of consolidation was determined as the only significant predictor for the overall survival in the final multivariable COX model (*p* < 0.001) (Table 2). Thus, the percentage of consolidation was defined to represent ‘AI score’. Interestingly, the radar charts in Fig. 3B showed that the GGO and consolidation patterns were symmetrically distributed, with an evident ’gravity gradient’ propensity to the lower area of the lungs, especially for the consolidation distribution.

### Comparisons of clinical performance between ‘IPF score’, ‘MDA5 score’ and ‘AI score’ model

**Figure 4:**
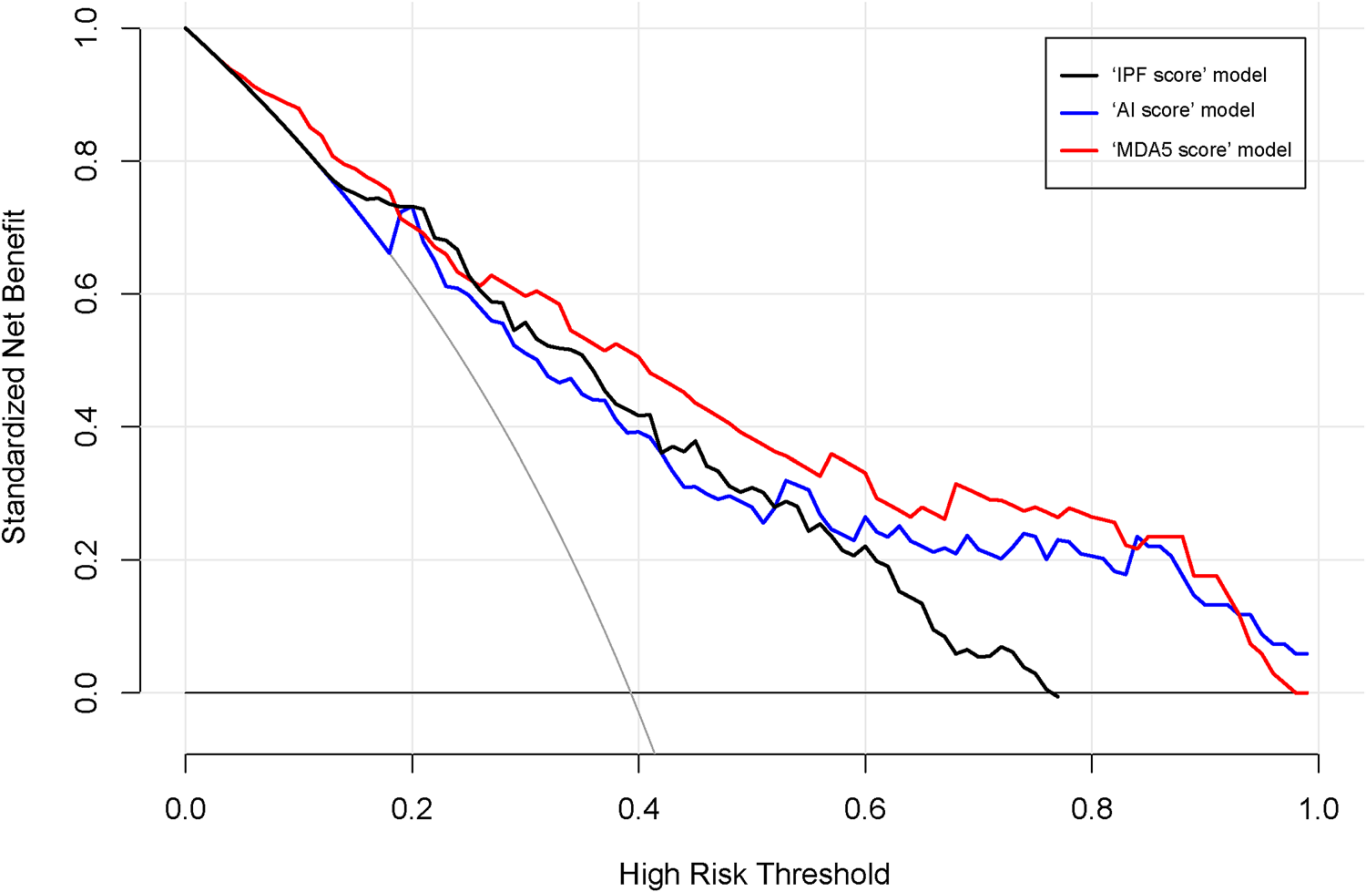
Decision curve analysis for ‘IPF score’, ‘MDA5 score’ and ‘AI score’ model. The concept of population net benefit (NB) is fundamental to decision curves (measured in the y-axis) and referred to classification accuracy of a model. Suppose high risk is defined as risk above some risk threshold R (x-axis); such high-risk patients are recommended an intervention. The NB of using the risk model was calculated by the true-positive rate, the proportion of cases with risk above risk threshold R; and the false-positive rate, the proportion of controls with risk above risk threshold R. The horizontal dotted line at NB = 0 mean a simple policy of no intervention to all patients (treat none); the gray curve in the plot depicted the NB of another simple policy: recommend the intervention to everyone regardless of risk. In our result, the ‘MDA5 score’ model (red line) had the highest net benefit compared to the others, almost across the full range of threshold probabilities.

The inter-observer consistency of the two visual scoring models was assessed with an ICC of 0.69 (95% CI 0.57–0.78) for ‘IPF score’ and an ICC of 0.93 (95% CI 0.89–0.96) for ‘MDA5 score’. Therefore, ‘MDA5 score’ attained a better inter-observer reproducibility. As a comparator, the detailed data of six domains for calculating ‘IPF score’ was presented in Supplementary table S2. Hereafter, the comparisons of model discrimination between ‘IPF score’, ‘MDA5 score’ and ‘AI score’ were shown in Table 3. Notably, ‘MDA5 score’ had the best performance with C-index values of 0.80 (95%CI 0.75–0.86) in the derivation dataset and 0.84 (95%CI 0.71–0.97) in the validation dataset, respectively. While, ‘AI score’ yielded C-index 0.78 (95%CI 0.72–0.84) for the derivation dataset and 0.77 (95%CI 0.64–0.90) for the validation dataset. Finally, the DCA further demonstrated that the ‘MDA5 score’ also presented with a higher overall net benefit than the other two models in terms of clinical applicability (Fig. 4).

**Table 3:** Comparison of the prediction performance of each model.

Scoring model	C-index [95%CI]	
	Derivation dataset (n = 116)	Validation dataset (n = 57)
‘IPF score’	0.78 [0.71–0.84]	0.78 [0.65–0.91]
‘MDA5 score’ *	0.80 [0.75–0.86]	0.84 [0.71–0.97]
Total GGO score + 2*total consolidation score		
‘AI score’	0.78 [0.72–0.84]	0.77 [0.64–0.90]
Percentage Of consolidation		

* ‘MDA5 score’ model performed significantly better than ‘IPF score’ model (p = 0.02).

C-index, concordance index; 95%CI, 95% confidence interval; IPF, idiopathic pulmonary fibrosis; MDA5, anti-melanoma differentiation-associated gene 5; GGO, ground-glass opacity; AI, artificial intelligence.

## Discussion

As a highly progressive disease, MDA5^+^ DM-ILD remains to be a big challenge despite of recent treatment advances^4, 14^. Several prognostic indicators of the disease had been reported involving respiratory physiology parameters, laboratory biomarkers, and radiology features^1, 6, 15, 16^. The current study focused on patients’ baseline pulmonary HRCT and attempted to quantitatively assess the disease in the regard of predicting six-month mortality.

Our study takes a step-forward from the previous visual scoring methods, and extensively evaluates the distribution and extent of three basic imaging components of MDA5^+^ DM-ILD, i.e., GGO, consolidation and fibrosis. In line with prior reports, our data confirmed that the presence of fibrosis or TBE in the context of GGO or consolidation, is not of predictive value on prognosis in MDA5^+^ DM-ILD^5, 10^. The probable explanation is that those fibrotic features are less common in this rapid progressive disease and likely to be presented, if it happens, in later stage instead of baseline. The same notion apparently holds true for the presence of PNM, which is a known severity indicator rather than a baseline predictor^17^.

The combination of the extent of GGO and consolidation was found to have the best yield in terms of outcome prediction, with the area of consolidation contributing more than GGO. The image ‘snapshot’ might reflect the dynamic transformation from GGO to consolidation as disease progresses, just like the imaging changes observed in severe COVID-19 patients^11, 12, 18^. A possible shared underlying mechanism of acute lung injury in the two diseases is a very intriguing question deserves further investigation. After all, the highly activated type I interferon pathway in MDA5^+^ DM-ILD which suggested a possible virus-triggered response has been postulated^19, 20^.

To apply AI algorithm-based quantitative imaging analysis in MDA5^+^ DM-ILD is a preliminarily yet novel attempt. The initial primary applicable population of this algorithm was pneumonia, or more specifically, COVID-19 disease. Of interest, our data suggested that this AI algorithm performs fairly well among MDA5^+^ DM-ILD patients. The performance might be further enhanced given more MDA5^+^ DM-ILD imaging data could be fed into its machine-learning processes.

The major limitation of our study was the single-center design. Although we presented a relatively large cohort for this rare disease and performed internal validation, large-scale multi-center external validation is mandatory before the CT scoring models being utilized in a clinical setting. Based upon this, the biases of different machine conditions, patient selection and treatment protocols could be taken into consideration and subjected to better control and adjustment. In addition, longitudinal analysis on the changes of ILD patterns over time remains untouched in the current study, which deserves further exploration.

In conclusion, we have shown that a GGO and consolidation-weighted CT scoring model, along with an AI algorithm, might serve as prognostic predictors for six-month mortality in MDA5^+^ DM-ILD. This might facilitate future clinical trial design and precision management for this tricky disease.

## Methods

### Patients

A retrospective cohort of hospitalized patients with MDA5^+^ DM-ILD was setup since April 2014 in our center. All patients initially fulfilled Bohan and Peter’s criteria for DM or Sontheimer’s criteria for clinically amyopathic dermatomyositis on admission^21, 22^, were re-evaluated and considered eligible as long as they also met the recent 239^th^ ENMC classification criteria for DM^23^. All patients were with imaging-confirmed ILD and positive anti-MDA5 antibody. ILD course was defined as time from the first abnormal pulmonary CT which revealed ILD changes to admission. Patients with ILD course > 3 months or with coexisting malignancy (within 3 years) or with pre-existing chronic obstructive pulmonary disease were excluded. The primary outcome was the six-month all-cause mortality since the time of admission.

A total of 173 eligible patients were enrolled and were further divided into two datasets. Patients admitted between April 2014 and December 2018 (n = 116) versus those admitted between January 2019 and January 2020 (n = 57), were defined as the derivation dataset and the validation dataset, respectively (Fig. 1).

Clinical data including age, gender, physical findings, respiratory function, treatment history and outcomes were obtained from medical records. The study was approved by the Shanghai Jiao tong University School of Medicine, Renji Hospital Ethnics Committee. The need to obtain informed consent was waived by the same committee. All methods performed in the study involving human participants were in accordance with the ethical standards of the Helsinki Declaration and its later amendments or comparable ethical standards.

### Measurement of autoimmune antibodies

The semi-quantitative detection of anti-MDA5 and other myositis specific antibodies (MSAs) was performed with EUROLINE Autoimmune Inflammatory Myopathies 16 Ag (IgG) (Euroimmun, Germany).

Quantification of anti-MDA5 antibody as confirmatory was conducted by the enzyme linked immunosorbent assay (ELISA). Firstly, purified recombinant MDA5 antigen (rMDA5) (Freezone Biotechnology co., LTD, Shanghai, China) diluted to 5 μg/mL in phosphate-buffered saline (PBS), was coated onto 96-well Microtiter plates (Maxisorp; Nunc, Rochester, NY, USA) overnight at 4 °C. The plates were washed twice with PBS and blocked with PBS containing 1% bovine serum albumin (BSA) and 5% sucrose overnight at 4 °C. Secondly, the serum samples were diluted at 1:101 in PBS containing 0.5% sodium chloride, 0.15% Tween 20, 0.2% BSA. Incubated for 30 min at room temperature. The plates were then washed four times with PBS containing 0.05% Tween 20 and incubated with Goat-conjugated anti-human IgG (PROMEGA, USA) diluted 1:60,000 in Conjugate Stabilizer (Thermo, USA). Finally, after incubation for 30 min at room temperature, the plates were washed 4 times and the bound antibodies were detected with the peroxidase substrate, 3, 3’, 5, 5’-tetramethylbenzidine. After incubation for 10 min at room temperature, the reaction was stopped by the addition of 0.5 N sulfuric acid. Absorbance at 450 nm (A) was measured, and unit values (IU/mL) were calculated from the following formula: 100 × (sample OD—blank OD) / (anti-MDA5-positive reference OD—blank OD). The cut-off level was set at 35 IU/ml.

### HRCT images acquisition and visual scoring

Patients underwent non-contrast pulmonary HRCT at the day around admission (median, 2 days; range, 1–6 days), using multidetector CT scanner (United Imaging, Shanghai, China; Siemens Healthineers, Forchheim, Germany). CT slice thickness was 1.0–1.5 mm at 10 mm intervals in the whole lungs.

All CT images were reviewed by two observers (YZ with 10-years’ experience and CZ with 5-years’ experience in chest HRCT imaging evaluation) who were blinded to patients’ outcome. Inter-observer variability was evaluated by Intraclass correlation coefficient (ICC). The results were agreed upon by consensus between the two observers.

For the previously reported IPF-based visual scoring method (‘IPF score’), HRCT findings were graded on a scale of 1–6 based on the classification system: 1, normal attenuation; 2, GGO without TBE; 3, consolidation without TBE; 4, GGO associated with TBE; 5, consolidation associated with TBE; and 6, honeycombing (Fig. 1)^8^. The overall ‘IPF score’ was calculated by summing the average score of six zones (upper, middle, and lower on both sides) as described; and was used as a comparator for the following analysis.

Three components, i.e. GGO, consolidation and fibrosis, were separately rated and recorded according to pulmonary involvement area of the five lobes (right upper, right middle, right lower, left upper and left lower lobes of the lung). The 0–5 scoring for GGO or consolidation at each lobe was adopted (0, no involvement; 1, ≤ 5% involvement; 2, 5 to < 25% involvement; 3, 25–49% involvement; 4, 50–75% involvement; 5, > 75% involvement). Similarly, the fibrotic change in each lobe was classified into 5 grades (0, no fibrosis; 1, interlobular septal thickening without honeycombing; 2, honeycombing < 25%; 3, 25–49%; 4, 50–75%; 5, > 75% of the lobe) as fibrosis score^9, 10^. The respective total score of each component (GGO, consolidation and fibrosis) was the sum of each lobe’s score and ranged from 0 (no involvement) to 25 (maximum involvement).

### AI algorithm-based CT quantitative analysis

The Digital Imaging and Communications in Medicine files of CT images were inputted and run on a software package named “CT Pneumonia Analysis” (syngo.via Frontier 1.0, Siemens Healthineers, Forchheim, Germany). The algorithm had been first trained on a large cohort of patients with various diseases, then fine-tuned with a cohort with abnormal patterns including GGO, consolidation, effusions, and masses, to improve the robustness of the lung segmentation over the involved areas. Based on 3D segmentations of lesions, lungs, and lobes, the AI algorithm automatically detected and quantified abnormal tomographic patterns commonly present in pneumonia, such as GGO and consolidation both globally and lobe-wise.

The percentage of total opacity (total lesions) as well as the percentage of consolidation (with a cutoff of CT value ≥ -200 Hounsfield unit) was directly calculated for the whole lung. Then by subtracting consolidation from total lesion, the percentage of GGO was obtained for further analysis.

### Statistical analysis

Clinical data were described and compared between the derivation and validation datasets by univariable analysis. The Mann–Whitney U test, Chi-square test and Fisher's exact test were conducted, as appropriate. Clinical features with > 5% missing data were excluded for analysis. Due to the retrospective observational design, no sample size calculation was performed in the current analysis.

Among the three visual scoring components, i.e. GGO, consolidation and fibrosis, variables significantly associated with outcome in the univariable analysis were subsequently included in the multivariable COX proportional hazards model. The derived β regression coefficients were used to construct a linear weighted scoring model, defined as ‘MDA5 score’. Likewise, the percentage of GGO and consolidation from AI algorithm based quantitative analysis were used to construct another weighted scoring model, defined as ‘AI score’.

The optimal cutoff value of CT score was identified by receiver operating characteristic curve analysis. The association between CT score and six-month survival were assessed by Kaplan–Meier survival plot and log-rank test.

Model discrimination of the ‘IPF score’, ‘MDA5 score’ and ‘AI score’ models were quantified and compared by the Harrell concordance index (C-index) with 95% confidence interval (CI). A decision curve analysis (DCA) was built to determine and compare the clinical usefulness of each model^24^. Significance was defined as *p* < 0.05.

Statistical analyses were performed by SPSS software version 25 (IBM Corp., Armonk, NY, USA), and R software version 3.6.1 (http://www.Rproject.org). All the R codes were available at Github (https://github.com/tomato08217/MDA5).

## Supplementary Information

Supplementary Table.

## Data Availability

The datasets used and/or analysed during the current study are available from the corresponding author upon reasonable request.
