# Supplementary material for: A novel CT scoring method predicts the prognosis of interstitial lung disease associated with anti-MDA5 positive dermatomyositis
Source: Sci Rep. 2021 Aug 23;11:17070. doi: 10.1038/s41598-021-96292-w (PMC8382835; doi:10.1038/s41598-021-96292-w)
Supplement: Supplementary file 1 — Supplementary Table. [file 41598_2021_96292_MOESM1_ESM.pdf]

Running head: Lung CT scoring for MDA5+DM-ILD

A novel CT scoring method predicts the prognosis of interstitial lung disease associated with anti-MDA5 positive dermatomyositis

Wenwen Xu, M.D.[1], Wanlong Wu, M.D.[1], Danting Zhang, M.M.[1], Zhiwei Chen, M.M.[1], Xinwei Tao, M.M.[2], Jiangfeng Zhao, M.M.[1], Kaiwen Wang, M.M.[1], Xiaodong Wang, M.D.[1], Yu Zheng, M.M.[3]*, Shuang Ye, M.D.[1]*

[1] Department of Rheumatology, Shanghai Jiao Tong University School of Medicine Affiliated Renji Hospital, Shanghai, China

[2] CT scientific collaboration, Siemens Healthineers, Shanghai, China

[3] Department of Pulmonology, Shanghai Jiao Tong University School of Medicine Affiliated Renji Hospital, Shanghai, China

Wenwen Xu, and Wanlong Wu contributed equally to this article as co-first authors.

Address correspondence to

Dr. Shuang Ye, Department of Rheumatology, Shanghai Jiao Tong University School of Medicine Affiliated Renji Hospital, No. 2000 Jiangyue Road, 201112, Shanghai, China, E-mail address: ye_shuang2000@163.com;

Dr. Yu Zheng, Department of Pulmonology, Shanghai Jiao Tong University School of Medicine Affiliated Renji Hospital, No. 2000 Jiangyue Road, 201112, Shanghai, China, E-mail address: drzhengyu@yahoo.com.

Supplementary Table S1: Comparison of baseline clinical features, treatment and outcomes between derivation and validation datasets.

**Characteristics**	Derivation dataset (n=116)	Validation dataset (n=57)	P-value
six-month mortality	47 (40.5)	21 (36.8)	0.764
**Demographic**			
Male sex	38 (32.8)	26 (45.6)	0.139
Age, years	50 [42-59]	52 [45-58]	0.408
DM course*, month	2 [2-4]	2 [2-3]	0.263
ILD course†, week	4 [2-8]	5 [3-8]	0.078
**Extrapulmonary symptoms**			
Fever	72 (62.1)	42 (73.7)	0.179
Heliotrope sign	101 (87.1)	46 (80.7)	0.381
Gottron sign	97 (83.6)	45 (78.9)	0.588
Skin ulcer	22 (19.0)	9 (15.8)	0.763
Dysphagia	16 (13.8)	10 (17.5)	0.673
**Respiratory function**			
FVC% <50%	47 (41.2)	30 (52.6)	0.211
PaO_2/FiO_2 <200	15 (12.9)	12 (21.1)	0.246
**Laboratory data**			
CRP, mg/L	3.7 [0.4-9.6]	4.8 [0-17.9]	0.387
ESR, mm/H	33 [15-47]	30 [14-51]	0.946
Serum ferritin, ng/mL	927 [385-1535]	1289 [561-2672]	0.054
LDH, U/L	318 [248-442]	346 [276-503]	0.153
Lymphocyte, 10^9/L	0.7 [0.5-1.1]	0.7 [0.3-0.9]	0.07
Ckmax, U/L	99 [43-279]	139 [38-338]	0.276
ALT, U/L	57 [35-101]	73 [44-122]	0.059
AST, U/L	54 [33-104]	63 [40-123]	0.201
CEA, ng/mL	6.3 [2.8-11.7]	7.6 [5.2-12.0]	0.064
Anti-Ro52 Ab positive	68 (58.6)	40 (70.2)	0.191
Anti-MDA5 Ab titer, RU/mL	181 [153-228]	185 [149-229]	0.943
**Treatment**			
Max dosage of MP, mg/d	120 [60-200]	80 [80-240]	0.979
MP pulse therapy	13 (11.2)	5 (8.8)	0.82
Exposure to IS‡			
1 IS	45 (38.8)	22 (38.6)	1
⩾2 IS	57 (49.1)	35 (61.4)	0.175
Exposure to pirfenidone or nintedanib	52 (44.8)	26 (45.6)	1

Data are presented as median [IQR] for continuous variables and number (frequency) (%) for categorical variables.

*DM course, time from the first symptom of dermatomyositis (DM) to admission;

†ILD course, time from the first abnormal pulmonary CT which revealed ILD changes to admission;
‡IS, immunosuppressant drugs, include cyclophosphamide, cyclosporine, tacrolimus, mycophenolate mofetil, tofacitinib, rituximab, basiliximab, and tocilizumab.
FVC%, forced vital capacity percentage of predicted; PaO2/FiO2, arterial oxygen/fraction of inspiration oxygen; CRP, C-reactive protein; ESR, erythrocyte sedimentation rate; LDH, lactate dehydrogenase; CKmax, maximum creatine kinase from disease onset to admission; ALT, alanine transaminase; AST, aspartate transaminase; CEA, carcinoembryonic antigen; MDA5, melanoma differentiation-associated protein 5; Ab, antibody; MP, methylprednisolone.

Supplementary Table S2: Comparison of six domains calculating for IPF score between two datasets with different outcome.

	Derivation dataset				Validation dataset				
	All (n=116)	Survivors (n=69)	Non-survivors (n=47)	p-value*	All (n=57)	Survivors (n=36)	Non-survivors (n=21)	p-value*	p-value†
Normal attenuation	74.8±18.0	81.8±13.9	64.7±18.7	<0.001	69.4±22.9	77.0±19.9	56.4±22.0	<0.001	0.14
GGO without TBE	16.6±13.2	12.8±10.6	22.4±14.7	<0.001	19.7±16.7	15.6±15.8	26.7±16.0	0.002	0.36
CON without TBE	7.0±5.9	4.4±3.4	10.9±6.6	<0.001	9.7±7.1	7.2±5.5	14.0±7.5	<0.001	0.01
GGO with TBE	1.0±3.0	0.6±2.2	1.6±3.8	0.07	0.6±1.6	0.2±0.5	1.3±2.5	0.001	0.68
CON with TBE	0.4±1.3	0.4±1.2	0.5±1.4	0.4	0.5±1.7	0.0±0.2	1.3±2.7	<0.001	0.33
honeycombing	0	0	0	1	0	0	0	1	1

Data are presented as mean ± SD for continuous variables.

***** A comparison between the survivors and non-survivors groups.

† A comparison between the derivation and validation datasets.

GGO, ground-glass opacity; CON, consolidation; TBE, traction bronchiectasis.
